# The evolution of the actin binding NET superfamily

**DOI:** 10.3389/fpls.2014.00254

**Published:** 2014-06-05

**Authors:** Timothy J. Hawkins, Michael J. Deeks, Pengwei Wang, Patrick J. Hussey

**Affiliations:** ^1^School of Biological and Biomedical Sciences, Durham UniversityDurham, UK; ^2^College of Life and Environmental Sciences, University of ExeterExeter, UK

**Keywords:** networked superfamily, actin cytoskeleton, actin binding proteins, membrane adaptors, evolution

## Abstract

The *Arabidopsis* Networked (NET) superfamily are plant-specific actin binding proteins which specifically label different membrane compartments and identify specialized sites of interaction between actin and membranes unique to plants. There are 13 members of the superfamily in *Arabidopsis*, which group into four distinct clades or families. NET homologs are absent from the genomes of metazoa and fungi; furthermore, in plantae, NET sequences are also absent from the genome of mosses and more ancient extant plant clades. A single family of the NET proteins is found encoded in the club moss genome, an extant species of the earliest vascular plants. Gymnosperms have examples from families 4 and 3, with a hybrid form of NET1 and 2 which shows characteristics of both NET1 and NET2. In addition to NET3 and 4 families, the NET1 and pollen-expressed NET2 families are found only as independent sequences in Angiosperms. This is consistent with the divergence of reproductive actin. The four families are conserved across Monocots and Eudicots, with the numbers of members of each clade expanding at this point, due, in part, to regions of genome duplication. Since the emergence of the NET superfamily at the dawn of vascular plants, they have continued to develop and diversify in a manner which has mirrored the divergence and increasing complexity of land-plant species.

## Introduction

The Networked (NET) proteins are a superfamily of plant-specific actin-binding proteins which localize simultaneously to the actin cytoskeleton and specific membrane compartments and are suggested to couple these membranes to the actin cytoskeleton in plant cells (Deeks et al., 2012). Metazoans utilize a variety of adaptor proteins, including α-actinin, spectrin, filamin, and FERM-domain proteins to produce specialized sites of interaction between membrane and actin. Notably, however, all of these protein families are absent from plants, despite actin-membrane interactions remaining critical for the plant cell with actin filaments dominating microtubules during organelle and endomembrane trafficking (Boevink et al., 1998; Kandasamy and Meagher, 1999; Van Gestel et al., 2002; Langowski et al., 2010). Evidence is accumulating that the plant cell employs analogous factors of its own, including those of the NET superfamily, to fulfill this role. In light of this plant specialization, it is rewarding to consider the evolutionary significance of the NET proteins and chart the development of the superfamily through plant evolution.

The founding member of the superfamily, NET1A, was originally identified as a 288 amino-acid fragment that labels a filamentous network during screening of an *Arabidopsis thaliana* cDNA (GFP)-fusion expression library. Residues 1–94 of this 288 aa region are sufficient to associate directly with actin filaments. This minimal actin binding region, referred to as the NET actin-binding (NAB) domain, represents a new actin binding motif unique to plants with no apparent primary sequence homology to previously identified actin binding domains. The NAB domain defines the NET superfamily, of which there are 13 members in the *Arabidopsis thaliana* proteome, ranging in size from 25 to 199 kDa. These proteins divide into four families based on the NAB domain sequence and the structural organization and length of the C termini. The C terminal region, beyond the NAB domain, is variable between families but within each family the members share several areas of conservation throughout this portion. Despite primary sequence differences, the C terminal domains of all NET proteins are predicted to take on a coiled-coil secondary structure which may provide an interface for protein-protein interactions with itself, other NETs or additional binding partners (Deeks et al., 2012).

## Results

### The NET superfamily and expression programmes

**Figure 1:**
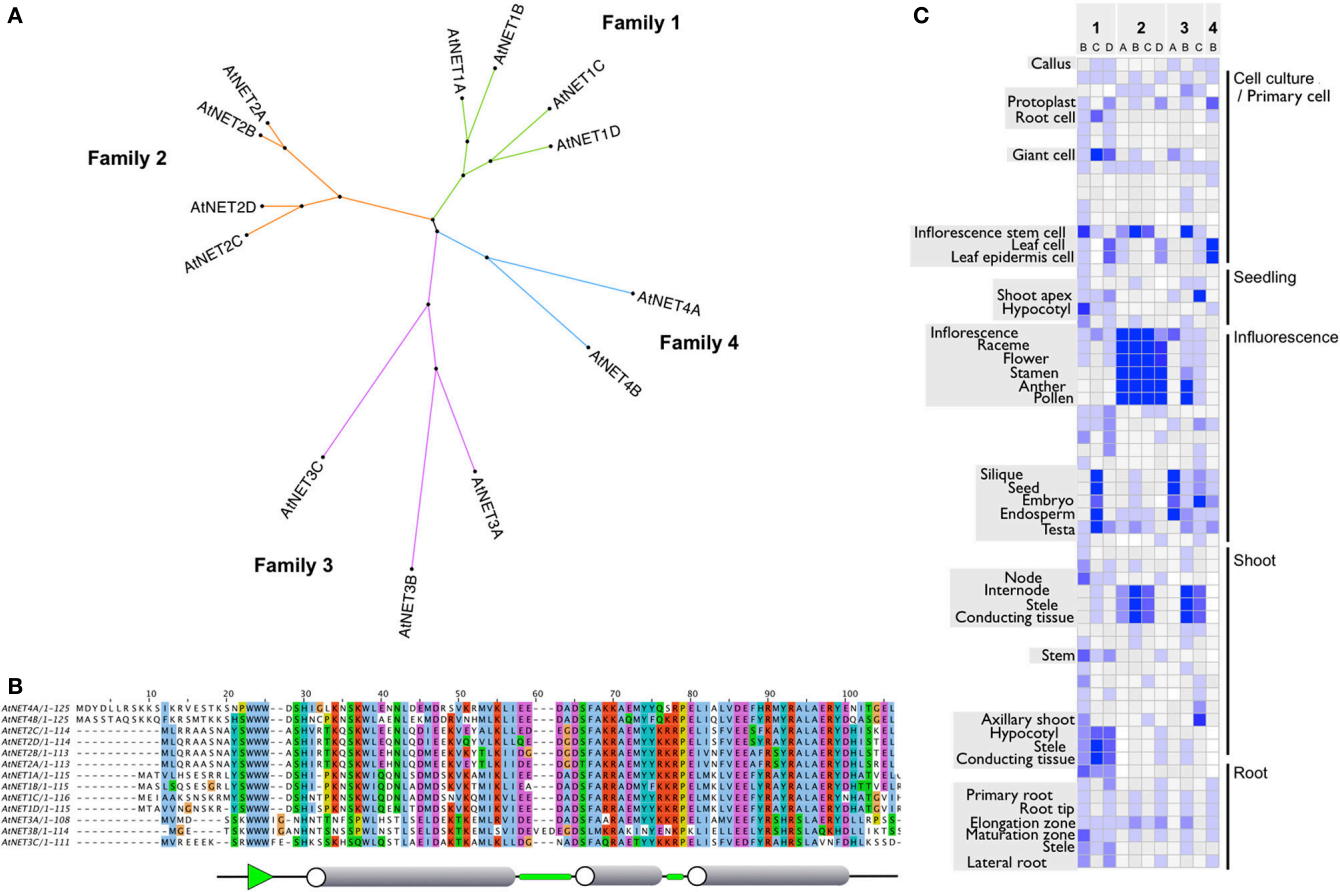
**(A)** Radial cladogram of the *Arabidopsis* NET protein superfamily. Protein sequences fall into four distinct clades or families. **(B)** NAB domain protein sequence alignment. ClustalX default residue coloring scheme based on physiochemical properties. Consensus secondary structure is shown below. Cylinders represent α helices, green sections show β turns, and green arrow represents β sheet. **(C)** Heatmap of NET family transcript level profiles. Adapted from publicly available DNA microarray data visualized with gene investigator.

The NET Superfamily separates into four phylogenetic families: 1–4 (Figure 1A and Deeks et al., 2012). There is high sequence conservation within the NAB domain across all of the four families, often with amino acid differences still representing residues of the same nature. In *Arabidopsis*, the NAB domain always starts with three conserved tryptophan residues, WWW, a motif whose worldwide web connection gives added significance to the NET family name (Figure 1B). The C terminal half of the domain is very highly conserved, more so than the N terminal. There are several residues which are identical in all NET NAB domains (W_15_W_16_W_17_,H_20_,S_25_,W_27_,L_32_,D_51_,A_57_,P_65_,R_79_, L_81_,A_82_) suggesting that, in addition to conserved motifs, these residues are likely to be essential for the structure of the domain and potentially its actin affinity. In general, NET3A and B have the most divergent NAB domain. Downstream of the core domain, there is further conservation between NET1 and 2 isoforms, indicating that these families may share a recent ancestor and/or common function. The predicted secondary structure of the domain includes three major α helices connected by β turns with the WWW motif predicted to form a β sheet. NET3B is unique in having an insertion in the sequence at turn 2.

Components of the NET superfamily show distinct expression profiles. Often, members of each family have primary zones in common but with subtle differences, where they are unique or predominant. Figure 1C gives an overview of the general expression profiles of the NET family and is adapted from publicly available DNA microarray data, visualized with gene investigator (Zimmermann et al., 2004) see Supplementary Figure 1 for complete tissue annotation. The NET1 family show peak expression levels in stele and conducting tissues of the root and hypocotyl with some lower expression in the elongation zone and tip. NET1C is the only NET1 family member which also has high expression in the tissues within the silique. Members of the NET2 family show peak expression within pollen and are almost exclusively found here. NET2B, however, does show a second peak within conductive tissue of the shoot. The NET3 family show more diversity. NET3A shows peak expression in the seed/endosperm/embryo and is almost exclusively found within these tissues of the silique. NET3B shows peak expression within pollen and the conductive tissue of the shoot and NET3C has peak expression at the shoot apex, embryo and hypocotyl. NET4B shows peak expression within the leaf with lower levels found in the root and silique.

NET1A is absent from this analysis as the probe used in the construction of the At22K chip not only covers the NET1A sequence but also the gene which resides next to it in the genome. We have previously shown, however, that a GUS reporter line for the NET1A promoter shows high levels of expression within the root. NET4A is not included as it is not represented on the chip. Again, we have demonstrated that the NET4AGFP fusion protein expressed under the control of the native NET4A promoter is expressed in the epidermis of the root elongation zone (Deeks et al., 2012).

### Evolution of the superfamily

**Figure 2:**
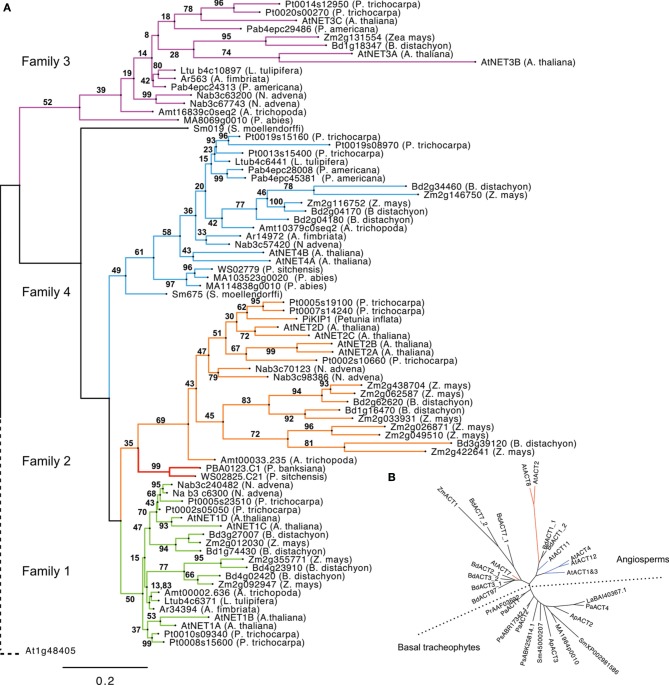
**(A)** Phylogeny of NET proteins calculated from the alignment of NAB domains. Branches are colored according to NET family. Gymnosperm 1/2 hybrid forms are separate in red. The phylogeny is rooted using the NAB-like N terminus of *Arabidopsis thaliana* protein At1g48405. **(B)**. Radial cladogram drawn from alignment of actin protein sequences from tracheophytes. The reproductive actins as defined by Kandasamy and Meagher (1999) are colored blue. Vegetative actins are red.

To chart the emergence and development of the NET proteins, the presence of the NET genes across a diverse range of species was assessed, ranging from the bryophyte *Physcomitrella patens* through many Tracheophyta to the crop Angiosperm *Zea Mays*. Tracheophyta genomes and ESTs surveyed were: Lycophytes (Spikemoss, *Selaginella moellendorffi*); Ferns (*Pteridium aquilinum, Adiantum capillus-v*eneris); Gymnosperms (*Picea abies, Pinus banksiana, Picea sitchensis*) and Angiosperms, (basal Angiosperm—*Amborella trichopod*a, Magnolids—*Aristolochia fimbriata*, *Persea americana*, Eudicots—*Arabidopsis thaliana, Populus trichocarpa*, and Monocots *Brachypodium distachyon*, *Zea mays*). No examples of NET proteins exist in non-plant genomes. (Figure 2A, see Supplementary Table 1 for a full list of NET orthologs).

Interestingly, members of the NET superfamily are completely absent from the genome of Bryophytes (mosses) and more ancient extant plant clades, which lack vasculature and can only be identified within the genomes of Tracheophytes (vascular plants). NET sequences are present in the genomes of all Tracheophyte species analyzed with the number of families represented increasing coincidentally with distinct stages of plant evolutionary complexity. Importantly, Bryophytes and Tracheophytes differ in the molecular mechanisms that couple the actin cytoskeleton to cell growth, for example genetic analysis of *P. patens* has shown that Bryophyte cell expansion requires the ARP2/3 complex, whereas Angiosperms appear considerably more resilient to equivalent genetic lesions (Harries et al., 2005).

NET proteins first emerge within the completed genome of the spikemoss, *Selaginella moellendorffii*. There are two examples present; one possesses a NAB domain that groups in phylogenetic analysis with the NET4 family and a second that does not, yet reciprocal BLAST searches indicate that the NAB domain of this protein most closely resembles that of NET4 proteins. This classification is further supported by analysis of the C-terminal portions of these proteins which exhibit the regions of homology found to be conserved between NET4 proteins in *Arabidopsis*. No examples of NET proteins of the remaining families are present. It does remain however that this more divergent NET4 like protein may be an orphan descendant of a distinct NET protein class.

The next major branch on the plant evolutionary tree of life is that of the ferns; here, information is limited. So far, whole-genome sequencing of a fern has not been economically feasible. However, despite this lack of a completed fern genome, some limited mining of NET sequences is still possible following the recent sequencing of the gametophyte transcriptome of bracken fern, *Pteridium aquilinum* (Der et al., 2011) and the fern EST database AcEST. Interrogation of the AcEST database revealed no NET sequences, however the much larger gametophye transcriptome contained a single example. This bracken NET sequence contains a NAB domain which shows most similarity to one of the two present in *Selaginella*, a NET4; although the sequence is truncated and as such not included in the ML tree. Ferns may, like *Selaginella*, only have NET proteins of family 4. However, without a complete genome and a transcriptome restricted to that of the gametophyte, it is reasonable that other NET examples may still exist in ferns.

**Figure 3:**
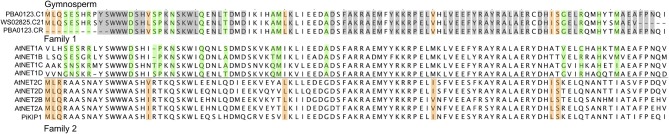
**Protein sequence alignment of Gymnosperm NET1/2 hybrid NAB domains with *Arabidopsis* NET1 and NET2 NAB domains**. Gray areas highlight those residues found in both NET1 and NET2 NAB domains. Green areas highlight those residues unique to NET1 NAB domains with orange areas highlighting residues unique to NET2 NAB domains.

Gymnosperms are a group of land plants, comprising Cycads, Ginkgo, Gnetophytes, and conifers, which first appeared more than 300 million years ago (Nystedt et al., 2013). There are several Gymnosperm EST databases and fortunately, very recently, the complete genome sequence of Norway Spruce has been completed (Nystedt et al., 2013). Considering sequences from both sources, it appears that Gymnosperms contain examples of the NET4 and NET3 families along with a NET protein which falls within the NET2 clade but possess a “hybrid” NAB domain with regions of homology characteristic of both family 1 and 2 NAB domains (Figure 3).

The next branch of the evolutionary tree to be surveyed is that of the Angiosperms, which differ from Gymnosperms primarily in their reproductive development and water-conducting xylem cells (Nystedt et al., 2013). The Angiosperm Eudicot, *Arabidopsis thaliana*, possesses examples of all four NET families, but how early in Angiosperm evolution did NET independent isoforms of the NET1 and 2 families emerge? To answer this question we need to consider a genome at the root of Angiosperm divergence. Until recently, the majority of sequenced Angiosperm genomes resided on just two limbs within the Angiosperm branch of the Tree of Life (Jansen et al., 2007; Moore et al., 2007; Soltis et al., 2008), yet many key Angiosperm innovations first appeared among the basal Angiosperm lineages (Soltis et al., 2002, 2005; Williams and Friedman, 2002; Friedman, 2006). Recently, the full genome sequence of *Amborella trichopoda* has been completed, a species identified as the single “sister species” to all other living flowering plants and is situated “between” Gymnosperms and all other Angiosperms (Soltis et al., 2008). Therefore, this genome was interrogated for the presence of NET proteins and revealed that *A. tricopoda* possesses NET proteins which fall into all four NET subclades, indeed a single example of each. This suggests that the emergence of the NET2 clade occurred early in the divergence of the Angiosperm lineage. The observation that NET1 and NET2 isoforms are found separately here, analogous to the two distinct families found in *Arabidopsis thaliana*, is of particular significance as NET2 proteins are pollen-specific NET isoforms and their emergence as individual proteins occurs at a point in plant evolution corresponding to the divergence of reproductive actin (Figure 2B) (Kandasamy et al., 2002). The timing of this specialization can also be seen in the diversification of ADF and Profilin families into forms adapted to interact with these two distinct subclades of actin isoforms (Kandasamy et al., 2007) which may suggest a co-evolution of the NET proteins with actin and known actin binding proteins. To fully substantiate such an hypothesis, a further rigorous comparison of evolutionary patterns in additional actin binding protein families would be required.

Modern Angiosperms in general can be classified into one of three sister clades: Monocots, Eudicots, and Magnolids. Inspection of the genomes of Monocot *Zea mays and Brachypodium distachyon* reveal that examples of all four NET families are also present here and that NET1s and NET2s are found separately, analogous to the two distinct families found in *Arabidopsis thaliana* and other Eudicots. In particular, maize has more NET2 isoforms than any other genomes analyzed.

Pertinently, even though three Magnolid EST databases were searched, no examples of NET2 isoforms can be identified, despite all three collections containing cDNA isolated from mature pollen. As described above, the basal Angiosperm genome of *Amborella trichopoda* does possess a recognizable NET2 sequence; yet in contrast to *Arabidopsis*, there is only a single example. NET2 sequences may be under-represented in these Magnolid transcriptomes and as no full Magnolid genome is currently available one cannot be certain, but it does appear to be a possibility that Magnolids have lost the progenitor NET2 found in basal Angiosperms, whereas Monocots and Eudicots have not only retained this clade but expanded it.

Further sequence analysis reveals that domain architecture is consistent across members of each clade from all species analyzed, although Gymnosperm NET4 examples are much larger than their counterparts in other species.

### NAB sequence conservation across species

When multiple sequence alignments of these NAB domains across species and complexity are considered, several features are conserved (Supplemental Figure 2). At the N terminus of NET3 isoforms from Monocot and Magnolid examples there is WWFD, which is also found in 3C (WWWFD as opposed to WWW). The region upstream of WWW appears to be less conserved when compared to other NET families. Magnolid and basal Angiosperm examples have an additional Serine residue before WWW, whereas Mono and Eudicots do not. The latter half of the domain is more highly conserved than the first part with the majority of sequences most similar to NET3C.

Interestingly, when one considers the NAB domain sequence of NET3 isoforms across species, the only example found to have the 3 amino acid insertion (VED) is *Arabidopsis* NET3B. This suggests that this occurred recently and uniquely in the genome of *Arabidopsis.* This sequence has been confirmed experimentally. The predicted secondary structure for this region of the domain is that of a loop, a structure where perhaps such an extension can be tolerated without abolishing the actin affinity of the NAB domain. Indeed, in GFP fusion experiments, AtNET3B does associate with actin filaments (data unpublished).

Cross species forms of the NET1 NAB domain are highly conserved throughout the domain. Upstream of WWW, these NAB domains have conserved RXYS. This YS preceding the WWW is a feature common with NET2 NAB domains, again pointing toward the shared ancestry of the NET1 and NET2 clades.

NET2 NAB domains are very conserved at the amino terminus, preceding the WWW motif. This sequence MLQRA is conserved in all Angiosperms examples and in many cases extends slightly beyond this (ASNAYSWWWASHIR), with the progenitor NET2 found in *Amborella trichopoda* only exhibiting small differences (E>G & A>SS). In fact, this sequence could be considered the defining feature of a NET2 type protein and may suggest that this sequence is important for the punctate localization at the pollen plasma membrane seen with NET2A.

Comparison of the NAB domain sequence of Gymnosperm NET1 like isoforms with *Arabidopsis* NET1 and NET2 proteins, reveals that it possesses features found to be common to both but also features which are unique to either form. This suggests that these Gymnosperm NAB domains represent a hybrid form predating a split which gave rise to the two independent forms found in Angiosperms and consistent with the functional divergence of reproductive actin (Figure 3). Specifically, the hybrid Gymnosperm NAB domain has part of the NET2 family defining MLQ sequence preceding the WWW motif, although the sequence following this region is predominately more characteristic of NET1. However, here there are still several residues which are found only in NET2 forms. The *Arabidopsis* NET1 to which these Gymnosperms seem most closely related is NET1D. If a complete fern genome sequence becomes available, it will be vitally important to identify if there are NET1 sequences within it to help pinpoint the emergence of NET1 isoforms.

### Expansion and multiplicity within subclasses

Following the emergence of new families, the number of members of each family rapidly multiplies in higher Angiosperms, specifically Monocots and Eudicots. In this analysis, we have primarily considered those complete genomes which can provide a full representation of NET isoforms present; however, it is intriguing that multiple Magnolid EST databases suggest that here such expansion is less prominent. The *Arabidopsis* genome contains numerous collinear clusters of genes which reside in large duplicated chromosomal segments, encompassing 60% of the genome. The sequence conservation between duplicated genes varies, as does the proportion of homologous genes in each duplicated segment, ranging from 20 to 47% (The *Arabidopsis* Genome Initiative, 2000).

**Figure 4:**
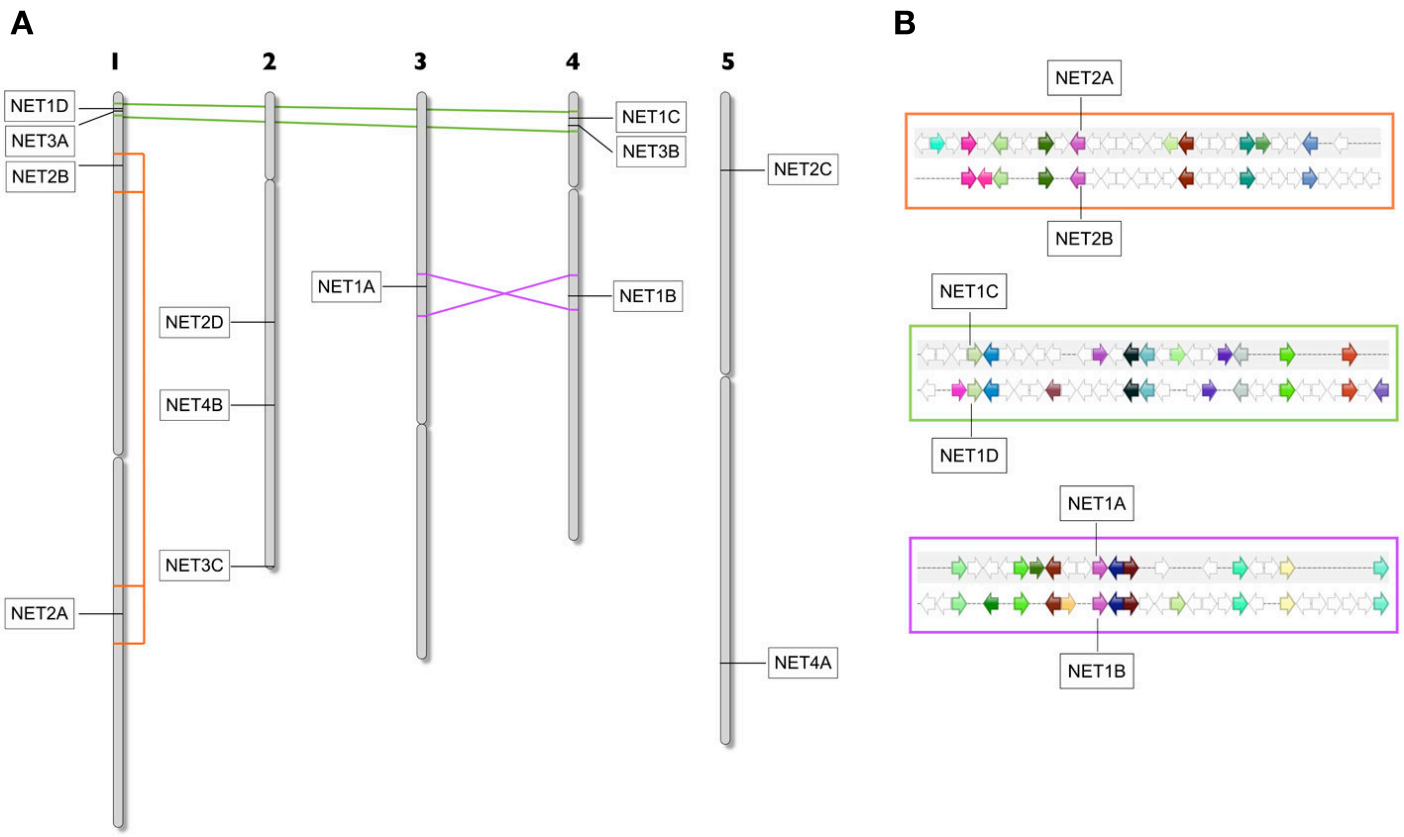
**(A)** *Arabidopsis* chromosome schematic showing areas of duplication which contain *Arabidopsis* NET genes. Duplication between Chr1 and 4 contain NET1D/NET3A and NET1C/NET3B. Duplication and inversion between Chr3 and 4 contains NET1A and NET1B. An internal Duplication in Chr1 contains NET2A and NET2B. **(B)** Zoomed area of multiplicon showing the position, conservation, and orientation of genes within duplications.

In order to ascertain whether such duplications could account for the family expansions observed in *Arabidopsis*, we performed an analysis of collinear clusters across all 5 chromosomes to identify those which contained NET protein isoforms (Figure 4). Here indeed, there are three significant genome duplications of regions in which NET genes reside. When the chromosomal locations of the *Arabidopsis* NET genes are plotted onto a chromosome schematic, it is striking that both chromosome 1 and 4 contain a NET1 isoform and a NET3 isoform in close proximity in the same orientation. Following collinear analysis, these genes are found to be within a duplicated region (multiplicon 23641) NET1C—NET1D and NET3A—NET3B. Secondly, there is a duplication and inversion between the mid regions of chromosome 3 and 4 (multiplicon 20748). Within this region are NET1A and NET1B. Thirdly, there has been an internal duplication within chromosome 1 (multiplicon 19672) leading to the duplication of NET2A and NET2B. Therefore, large scale comparatively recent genome duplications can, in part, account for expansion within NET families in *Arabidopsis*. Further analysis of chromosomal localities of NET isoforms in other Angiosperms, including Monocots, suggests that similar duplications have also occurred here. Additionally, the tandem duplication of NET coding sequences in the genome of maize may account for the large NET2 family found in this species.

In some cases, there are duplicated segments where the NET gene is not present on both copies of the duplication, for example both NET2C and NET4A reside in regions which have been duplicated within chromosome 5 but in both cases the gene is absent from the other copy. One explanation is that one copy has been lost, which suggests that most of the remaining *Arabidopsis* NET sequences have been maintained by natural selection.

This expansion in families may represent the evolution of additional unrecognized subclasses or diversification within the subclasses to generate isoforms that function in particular developmental, environmental, or physiological contexts as has been suggested for RAB GTPases (Rutherford and Moore, 2002). Indeed, inspection of microarray data does suggest that this is likely to be the case for the NET superfamily, although in some cases functional redundancy may also be evident.

## Discussion

**Figure 5:**
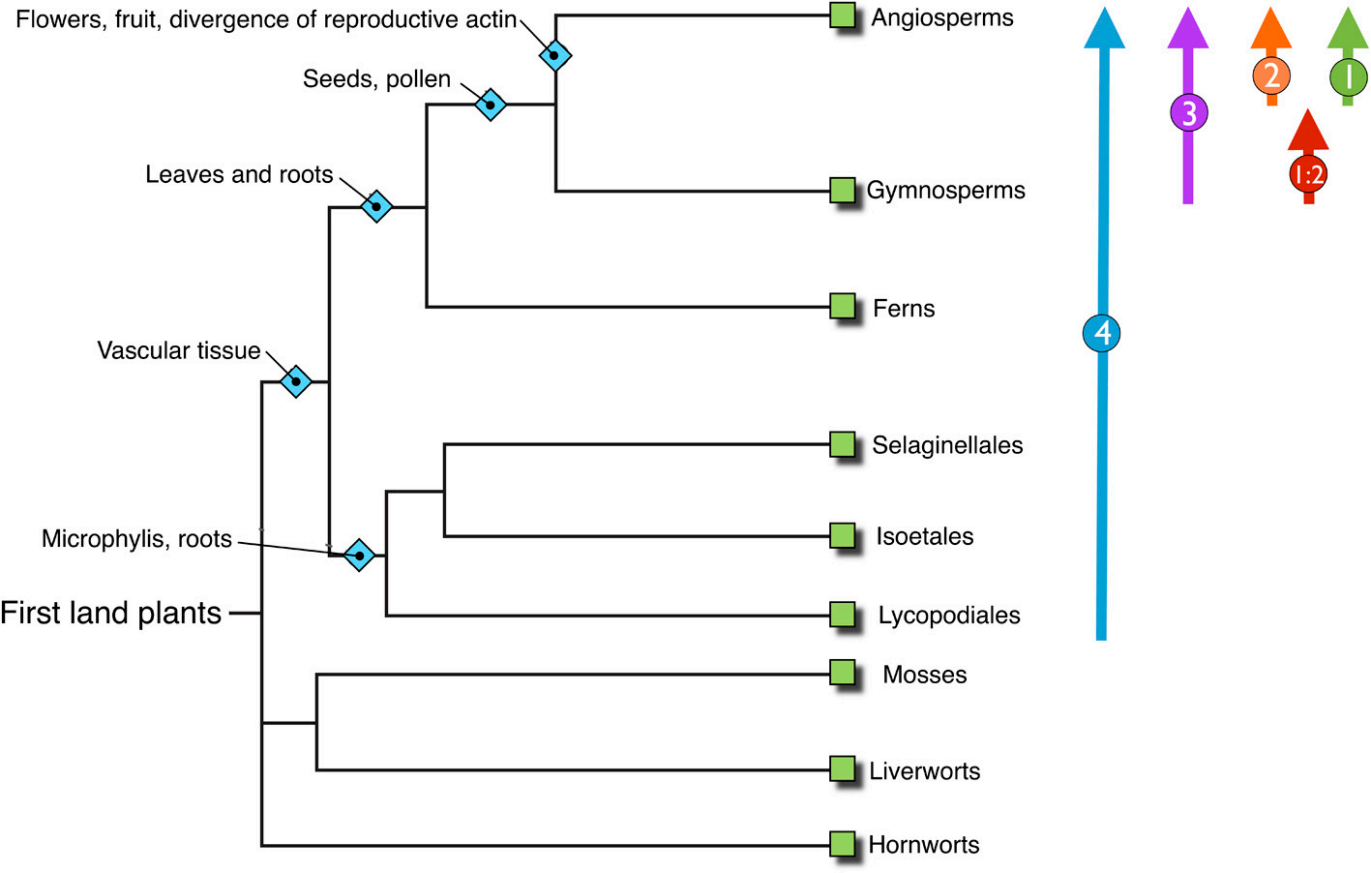
**Overview phylogenetic tree of land plants, inferring the progress of plant evolution**. NET families identified within those genomes are shown on the right illustrating the emergence and development of the NET family concurrent with steps in plant evolution and complexity.

Our analysis reveals the emergence and development of the NET superfamily through plant evolution. Significantly NET proteins are plant specific, with no examples found within the genomes of metazoa or yeast and are also absent from nonvascular plants including Bryopytes, first emerging in Sellaginella at the beginning of the vascular plant linage. From here the superfamily have continued to develop and diversify in a manner which has mirrored the divergence and complexity of plant species through evolution (Figure 5). Importantly a significant proportion of the NAB domain is conserved not only within all families but also across evolutionary diverse species. Strikingly several residues are identical in every NET protein identified. This suggests that these residues must be essential in providing surfaces, moieties or conferring the conformation required for actin binding. The differences may represent different affinities for F-actin, a preference for binding different actin isoforms or provide family specific control regions.

When considering the emergence of NET proteins in Sellaginella, it is notable that the Lycophytes represent a critical development in the evolutionary complexity of land plants, signifying the onset of vasculature development, with its origins dating as far back as the late Silurian/early Devonian period (Banks, 2009). A particular difference between vascular and nonvascular plants is the morphological complexity of the sporophyte generation; its function being to produce haploid spores, which in non-seed plants represent the principal method of reproductive dispersal. The very first land plant sporophyte was extremely basic, constituting a short, rootless cylinder complete with terminal sporangium (Kenrick and Crane, 1997). Therefore, in order to increase the efficiency of reproductive dispersal, one method would be to increase the height of the sporophyte, which correspondingly requires the evolution of specialized tissues facilitating the transport of water, nutrients and hormones, resistant to the effects of increased wind speed and gravitational forces (Raven, 1993; Banks, 2009).The co-occurrence of NET proteins with the development of vasculature suggests that NET proteins may have supported the changes observed in this step in plant complexity including transport tissues and resistance to increased mechanical, gravitational and osmotic pressures. It is tempting to speculate that perhaps a rigid structure surrounding the vacuole is beneficial in the tonoplast membrane to adapt to increases in turgor pressure required for land plant structural integrity, in particular in those cells at the surface of the tissue, the epidermis, where NET4A is found to be expressed.

Furthermore, there may be added significance to the fact that it is the NET4 class which emerge first and specifically at this point. Plant cells predominantly utilize actin microfilaments for the spatial regulation of their major membrane components, including the ER (Sheahan et al., 2004; Runions et al., 2005), Golgi (Boevink et al., 1998; Nebenfuhr et al., 1999), and importantly, the Vacuole (Ovecka et al., 2005; Higaki et al., 2006). Actin filaments colocalize with the vacuole membrane and following the breakdown of actin microfilaments by anti-actin agents vacuoles are seen to deform, fragment and lose their dynamics (Kutsuna et al., 2003; Ovecka et al., 2005). However, in direct contrast to plant cells, recent studies in *Physcomitrella patens* have demonstrated that here microtubules are required in maintaining the structure and distribution of the vacuole rather than actin (Oda et al., 2009). In Physcomitrella, microtubules and vacuolar membranes co-localized with elongating microtubules appearing to “tug” vacuolar membranes. Furthermore, actin depolymerization agents had little effect on vacuolar morphology whereas the microtubule depolymerization herbicide, oryzalin, clearly affected the vacuolar structures (Oda et al., 2009). These findings suggest the possibility of a divergence in the regulatory system of vacuolar structures by the cytoskeleton during land plant evolution. AtNET4A decorates actin filaments which lie upon the surface of the vacuole at the tonoplast membrane where they may be an adaptor responsible for linking the two, possibly aiding in the encaging of the vacuole (Deeks et al., 2012). It is notable that NET proteins, and in particular the tonoplast associated NET4 isoform, emerge at a point in plant evolution where there has been a shift to actin as the predominant regulator of vacuole structure and hence the requirement for novel factors to co-ordinate or link actin and the vacuole. It is tempting to speculate that here the NET superfamily emerge to fill this role.

Further on in plant evolution, the genome of the more complex Gymnosperms contain three NET isoforms, NET4, NET3 and a unique form of NET2 whose NAB domain is a hybrid NET1/2 form, sharing several features unique to 1 and 2 isoforms, including the upstream NET2 sequence MLQ. We have previously shown that an *Arabidopsis* member of the NET1 family, NET1A, is localized to the plasma membrane and particularly enriched at the plasmodesmata (PD) in root cells (Deeks et al., 2012). PD occur in all higher plants (Cook and Graham, 1999); indeed, the most closely related examples of Charophycean green algae from which Embryophytes evolved also possess PD, although the majority of extant Charophyceae do not. Studies have suggested a minimum of two and possibly more independent origins of PD in the Viridiplantae at the algal level and Heterokontophyta (Raven, 1997) Interestingly though, algal PDs differ in structure from that of higher plants with the absence of the desmotubule (Cook et al., 1997; Raven, 1997; Cook and Graham, 1999). It is considered that PD in Bryophytes and all vascular plants are comparable (Cook and Graham, 1999; Raven, 2005), although differences in the frequencies and development of PDs have been seen between ferns and Angiosperms within the shoot apical meristem (Imaichi and Hiratsuka, 2007; Jones, 1976; Cooke et al., 1996; Ehlers and Kollmann, 2001). Therefore, evolutionary development of PD significantly predates the emergences of NET1 isoforms; however, these forms may appear at a point where the structural complexity of the PD has advanced and here, new components are required to maintain this structure and assist in its functionality. The NET1 and 2 families are first seen as independent families at the divergence of Angiosperms, with the emergence of the pollen specific NET2 family at a point which corresponds to the divergence of pollen and vegetative actin isoforms.

The growth of the NET family can be compared to the sequence in which major terrestrial plant groups have appeared in the fossil record. Each new group has successfully competed against ancestral clades to dominate the land. These successes are attributed to increasing complexity and sophistication in cells and tissues that enhance reproduction, transpiration, nutrient transport, and plant structural integrity. From the pattern of NET diversification, it is tempting to speculate that the NETs have somehow contributed to driving these adaptations; however, this suggestion raises several broader questions including: Do other proteins that shape cell architecture show similar complexity-associated patterns of diversification across plants and other major groups? and does such an interpretation rely too heavily on considering plant evolution as a linear sequence of events?

This expansion of sub-types for cytoskeletal proteins is not true for every super-family. Bryophytes and Lycophytes contain three very distinct classes of formins, yet Angiosperms have retained only two classes (Grunt et al., 2008). Diversity has been lost along the journey as well as gained. The NETs are not, however, a single isolated example of diversification as the recently identified DUF593 super-family of myosin cargo receptors follow a similar trajectory of increasing subfamily variety (Peremyslov et al., 2013).

Considering plant evolution as a linear sequence misses the ever-branching tree of speciation and adaptation where most spurs are dead-ends and only occasional outgrowths give rise to the living examples from which we can collect molecular data. This pruning through fitness and bad luck (such as mass extinctions) occurs at the smaller scale of gene families and has perhaps led to the loss of NET2 within the extant mangolids.

Inevitably, our sample of genomic data is biased toward the plant species we find most valuable and the species with compact genomes that can be sequenced most efficiently. In this study, we have been limited by the absence of a completed fern genome, likely because of the costs incurred by their complex karyotype (Barker and Wolf, 2010). More high-quality data from many extant species are needed, including complete genome sequences, molecular cellular phenotyping and expression data that shows precisely where in time and space new genetic innovations are exploited. It will then be possible to realize one of the key aims of evolutionary-developmental studies: to make a major contribution to the functional analysis of novel proteins.

## Materials and methods

### Phylogenetic and domain analysis

*Arabidopsis thaliana* NET sequences were identified by screening the TAIR and Genbank sequence databases using the BLASTP and TBLASTN algorithms (Altschul et al., 1997). To identify NET homologs in other species, BLASTP and TBLASTN algorithms were used to screen: non-redundant and EST databases of Genbank; Norway spruce genome (http://congenie.org/); *Selaginella* genome (http://genome.jgi-psf.org/) Amborella genome http://www.amborella.org/; Magnolid EST databases, ancestral Angiosperm genome project (http://ancangio.uga.edu/); Fern EST databases AcEST (http://togodb.dbcls.jp/acest); and braken gametophyte unigenes at NCBI. Further BLAST analysis was conducted using the resources available at phytozome (http://www.phytozome.net/). Reciprocal TBLASTN screening of the TAIR database was used to validate sequences.

Multiple alignments were assembled in ClustalX (Larkin et al., 2007) Manual adjustment and cropping of multiple alignments were made using CINEMA (Parry-Smith et al., 1997) and exported as graphics using Jalview (Waterhouse et al., 2009). Secondary structure prediction of *Arabidopsis* NAB domains was performed by Jpred3 (Cole et al., 2008). The Arabidopsis NET family tree was generated from multiple alignments by applying the neighbor-joining method to a bootstrapped dataset with 1000 replicates (Saitou and Nei, 1987).

The Maximum likelihood method was chosen for the NET evolution phylogenetic tree as this method has been identified as one of the most robust optimality criterion (Felsenstein, 1981, 2004; Swofford et al., 2001) Maximum Likelihood trees were calculated in the MetaPIGA software package (Helaers and Milinkovitch, 2010), using stochastic heuristics for large phylogeny inference with the Metapopulation Genetic Algorithm (metaGA) (Lemmon and Milinkovitch, 2002). MetaGA is an evolutionary computation heuristic in which several populations of trees exchange topological information which is used to guide the Genetic Algorithm (GA) operators for much faster convergence. The MetaGA algorithm was chosen as it resolves the problems inherent to classical GAs including the need to choose between strong selection (speed) and weak selection (accuracy) by maintaining high inter-population variation even under strong intra-population selection. Furthermore, MetaGA generates branch support values that approximate posterior probabilities. Dataset quality control included testing for identical sequences and excessively ambiguous or excessively divergent sequences and automated trimming of poorly aligned regions using the trimAl algorithm. Within MetaPIGA statistical methods, including Likelihood Ratio Test, Akaike Information Criterion, and Bayesian Information Criterion were used to select the amino-acid substitution model that best fitted the data. MetaPIGA calculations were stopped when the mean relative error of 10 consecutive consensus trees stayed below 5% using trees sampled every 5 generations or the Likelihood stopped increasing after 200 iterations. Trees were drawn and exported as graphical files from FigTree (Andrew Rambout, University of Edinburgh).

### Genome analysis

The comparative genomics tools available at PLAZA, (Bel et al., 2012) (http://bioinformatics.psb.ugent.be/plaza/) including WGDotplot, were used to identify *Arabidopsis* genome duplications and the presence of NET proteins within these regions.

## Author contributions

Timothy J. Hawkins conducted the analysis; Michael J. Deeks contributed additional bioinformatics data; Pengwei Wang contributed additional experimental data; Timothy J. Hawkins, Michael J. Deeks, and Patrick J. Hussey wrote the manuscript.

### Conflict of interest statement

The authors declare that the research was conducted in the absence of any commercial or financial relationships that could be construed as a potential conflict of interest.
