# Supplementary material for: The evolution of the actin binding NET superfamily
Source: Front Plant Sci. 2014 Jun 5;5:254. doi: 10.3389/fpls.2014.00254 (PMC4046492; doi:10.3389/fpls.2014.00254)
Supplement: Supplementary file 1 [file Presentation1.ZIP › Supp Table 1.PDF]

Family 1		Family 2		Family 3		Family 4	
Ar34394	A. fimbriata	AtNET2D	A. thaliana	Ar563	A. fimbriata	Ar14972	A. fimbriata
AtNET1D	A. thaliana	AtNET2C	A. thaliana	Amt16839	A. trichopoda	AtNET4B	A. thaliana
AtNET1C	A. thaliana	AtNET2B	A. thaliana	AtNET3C	A.thaliana	AtNET4A	A. thaliana
AtNET1B	A. thaliana	AtNET2A	A. thaliana	AtNET3A	A.thaliana	Amt10379	A. trichopoda
AtNET1A	A. thaliana	Amt00033.235	A. trichopoda	AtNET3B	A.thaliana	Bd2g04170	B distachyon
Amt00002.636	A. trichopoda	Bd2g62620	B. distachyon	Bd1g18347	B. distachyon	Bd2g34460	B. distachyon
Bd3g27007	B. distachyon	Bd1g16470	B. distachyon	Ltu b4c10897	L. tulipifera	Bd2g04180	B. distachyon
Bd1g74430	B. distachyon	Bd3g39120	B. distachyon	Nab3c63200	N. advena	Ltub4c6441	L. tulipifera
Bd4g23910	B. distachyon	Nab3c70123	N. advena	Nab3c67743	N. advena	Nab3c57420	N advena
Bd4g02420	B. distachyon	Nab3c98386	N. advena	MA8069g0010	P. abies	MA103523g0020	P. abies
Ltub4c6371	L. tulipifera	Nab3c6300	N. advena	Pab4epc29486	P. americana	MA114838g0010	P. abies
PBA0123.C1 P. banksiana				Pab4epc24313	P. americana	Pab4epc28008	P. americana
		KIP1	P. inflata	Pt0014s12950	P. trichocarpa	Pab4epc45381	P. americana
WS02825.C21 P. sitchensis				Pt0020s00270	P. trichocarpa	WS02779	P. sitchensis
Nab3c240482	N. advena	Pt0005s19100	P. trichocarpa	Zm2g131554	Zea mays	Pt0019s15160	P. trichocarpa
Pt0005s23510	P. trichocarpa	Pt0007s14240	P. trichocarpa			Pt0019s08970	P. trichocarpa
Pt0002s05050	P. trichocarpa	Pt0002s10660	P. trichocarpa			Pt0013s15400	P. trichocarpa
Pt0010s09340	P. trichocarpa	Zm2g438704	Z. mays			Sm675	S. moellendorffi
Pt0008s15600	P. trichocarpa	Zm2g062587	Z. mays			Sm019	S. moellendorffi
Zm2g012030	Z. mays	Zm2g033931	Z. mays			Zm2g146750	Z. mays
Zm2g355771	Z. mays	Zm2g026871	Z. mays			Zm2g116752	Z. mays
Zm2g092947	Z. mays	Zm2g049510	Z. mays			*Pab4epc55650	P. americana
*Nab3c41185	N. advena	Zm2g422641	Z. mays			*ptaqESTirc67056	P. aquilinum
*MA10205g0010 P. abies						*WS02776	P. sitchensis

Supplementary Table 1. NET protein orthologs found in genome and EST databases. *Selaginella moellendorffi*, *Pteridium aquilinum, Adiantum capillus-*veneris, *Picea abies, Pinus banksiana, Picea sitchensis*, *Amborella trichopoda, Nuphar advena, Liriodendron tulipifera, Aristolochia fimbriata*, *Persea americana, Petunia inflata*, *Arabidopsis thaliana, Populus trichocarpa*, *Brachypodium distachyon*, *Zea mays*.

* These sequences were not used within the ML tree due to quality control procedures. These examples have been assigned family groupings based on BLAST similarity scores and position in Neighbour joining trees.
