# Supplementary figures and images for: The evolution of the actin binding NET superfamily
Source: Front Plant Sci. 2014 Jun 5;5:254. doi: 10.3389/fpls.2014.00254 (PMC4046492; doi:10.3389/fpls.2014.00254)

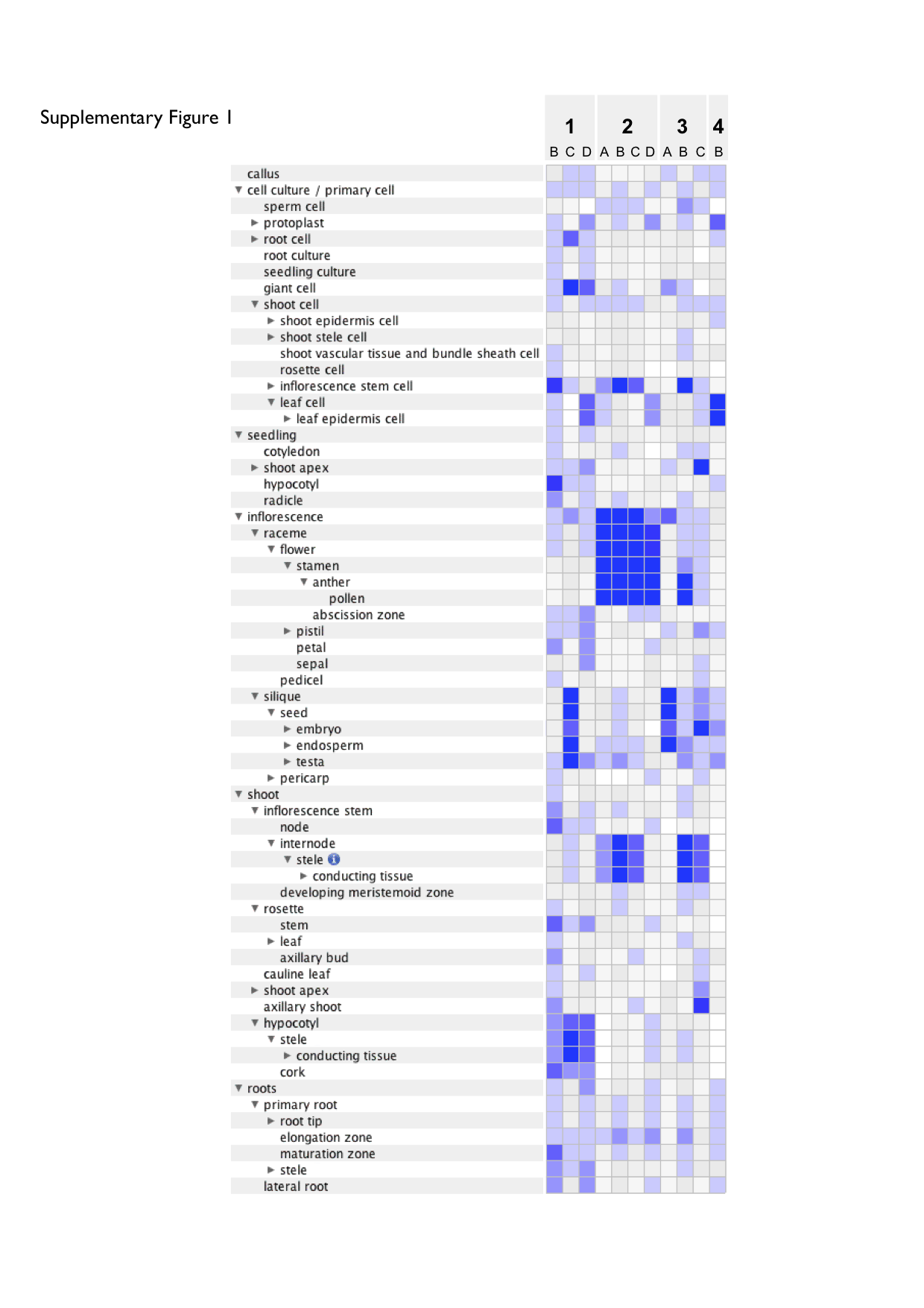

Supplement: Supplementary file 1 [file Presentation1.ZIP › Supp Figure 1.TIFF]

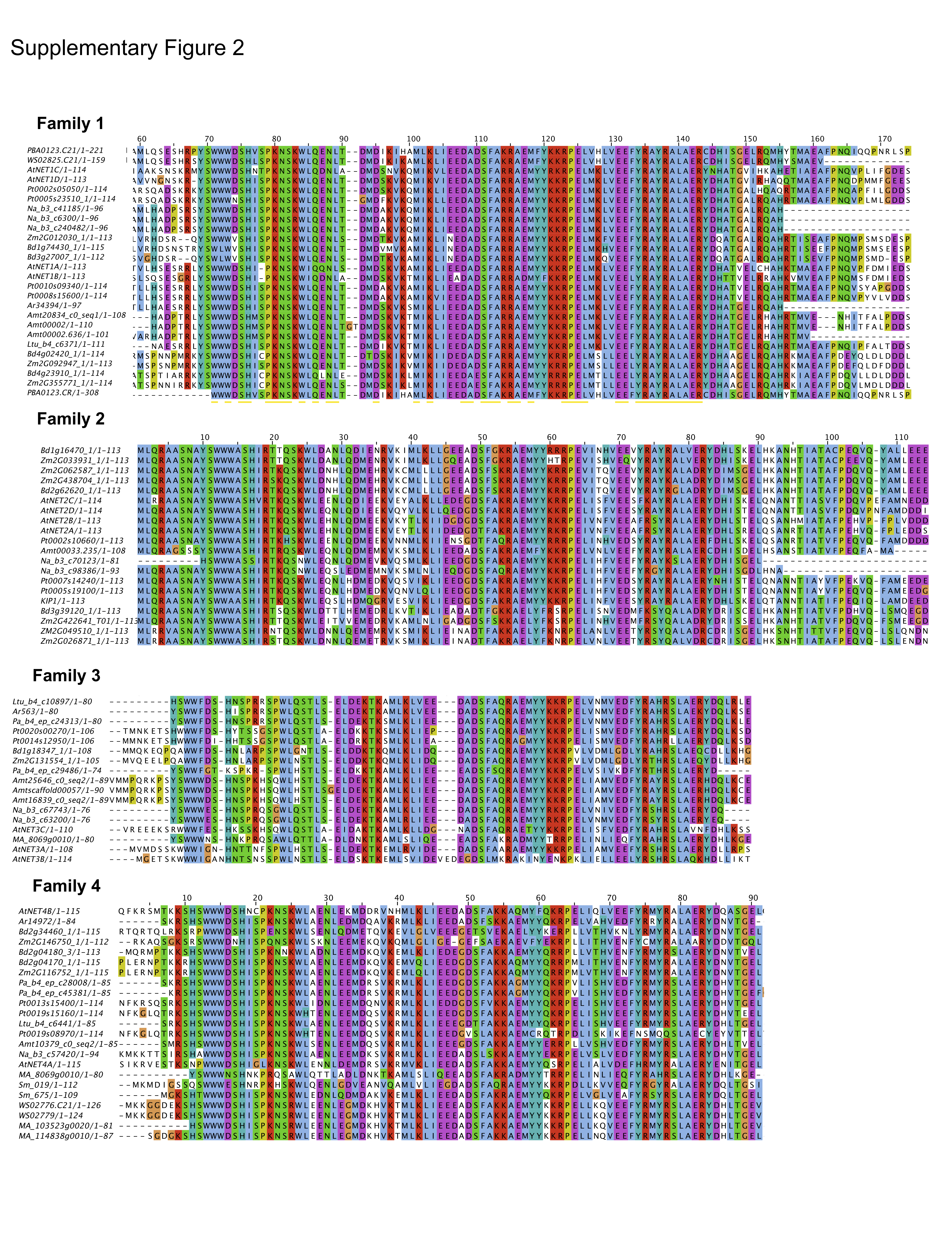

Supplement: Supplementary file 1 [file Presentation1.ZIP › Supp Figure 2.TIFF]
